# COMPARTMENTS: unification and visualization of protein subcellular localization evidence

**DOI:** 10.1093/database/bau012

**Published:** 2014-02-25

**Authors:** Janos X. Binder, Sune Pletscher-Frankild, Kalliopi Tsafou, Christian Stolte, Seán I. O’Donoghue, Reinhard Schneider, Lars Juhl Jensen

**Affiliations:** ^1^Structural and Computational Biology Unit, European Molecular Biology Laboratory (EMBL), 69117 Heidelberg, Germany, ^2^Bioinformatics Core Facility, Luxembourg Centre for Systems Biomedicine (LCSB), University of Luxembourg, 4362 Esch-sur-Alzette, Luxembourg, ^3^Department of Disease Systems Biology, Novo Nordisk Foundation Center for Protein Research, Faculty of Health and Medical Sciences, University of Copenhagen, 2200 Copenhagen, Denmark, ^4^CSIRO Computational Informatics, Sydney, NSW 2113 Australia and ^5^Garvan Institute of Medical Research, Sydney, NSW 2100, Australia

## Abstract

Information on protein subcellular localization is important to understand the cellular functions of proteins. Currently, such information is manually curated from the literature, obtained from high-throughput microscopy-based screens and predicted from primary sequence. To get a comprehensive view of the localization of a protein, it is thus necessary to consult multiple databases and prediction tools. To address this, we present the COMPARTMENTS resource, which integrates all sources listed above as well as the results of automatic text mining. The resource is automatically kept up to date with source databases, and all localization evidence is mapped onto common protein identifiers and Gene Ontology terms. We further assign confidence scores to the localization evidence to facilitate comparison of different types and sources of evidence. To further improve the comparability, we assign confidence scores based on the type and source of the localization evidence. Finally, we visualize the unified localization evidence for a protein on a schematic cell to provide a simple overview.

**Database URL:** http://compartments.jensenlab.org

## Introduction

Determining the subcellular localization of a protein is a key step toward understanding the cellular function of a protein. Therefore, knowledge on protein subcellular localization is manually curated by UniProtKB (1) and model organism databases such as MGI (2), SGD (3), FlyBase (4) and WormBase (5). These databases also integrate data from cDNA tagging projects (6–8), proteomics-based experiments (9, 10) and microscopy-based high-throughput localization studies (11–14). However, an ongoing effort like the Human Protein Atlas (HPA) (15) is only partially integrated in UniProtKB, and thus needs to be treated separately to obtain a comprehensive view of the currently available experimental data on localization.

Despite the huge efforts by curators working for the databases mentioned above, it is impossible to fully keep up with the ever-growing literature. Thus automatic text-mining methods can complement human curators. Several text-mining methods have been developed to automatically extract localization information from the biomedical abstracts (16–18).

Even if one combines curated knowledge, primary experimental data and text mining, there will still be many proteins with little or no information on their localization. Fortunately, the protein sequence itself contains clues to where the protein is localized, such as protein sorting signals, the amino acid composition and sequence homology (19). Examples of sequence-based subcellular localization prediction methods are BaCelLo (20), LOCtree2 (21), PSORT (22) and YLoc (23, 24).

As these different types and sources of information are complementary, it is important to take them all into account. However, this is not trivial. The databases and experimental data sets come in various file formats and use different identifiers/names for the same proteins and cellular compartments. The sequence-based prediction methods have different web interfaces, the prediction outputs consist of scores that are not directly comparable and local installation of the software is generally required for genome-wide analyses. It is thus difficult and time-intensive to collect and evaluate the evidence pertaining to the subcellular localization of a protein of interest, not to mention for a large number of proteins.

Several databases have attempted to address this data integration challenge. An early effort was DBSubLoc (25), which integrated annotations from knowledge bases such as UniProtKB and the major model organism databases. Manual annotations were complemented by sequence-based predictions in eSLDB (26) and further by experimental data sets in LOCATE (27), locDB (28) and SUBA3 (29). The most recent versions of the first three of these resources (DBSubLoc, eSLDB and LOCATE) are >5 years old, and thus, they cannot be considered to reflect the current evidence. The last two resources (locDB and SUBA3) have been updated within the past 2 years; however, between them these two resources cover only human and *Arabidopsis thaliana* proteins. Whereas these resources are, or were, collecting evidence from a variety of sources in a single database, they generally do not address the challenge of putting the different types of evidence on a common confidence scale. An exception is the *A. thaliana* resource SUBA3, which assigns an overall confidence score; however, it is difficult for the user to trace these scores back to their origin.

We have developed an automatically updated web resource to be able to provide up-to-date information on the subcellular localization of proteins from the major eukaryotic model organisms. In addition to integrating manually curated annotations, experimental data and predictions, we use automatic text mining to extract associations from the biomedical literature. Unlike earlier resources, we address the challenge of making evidence comparable across types and sources by introducing a unified confidence scoring scheme. To further shield users from the heterogeneity of the many evidence sources, we map all localization evidence onto Gene Ontology (GO) terms and visualize the combined results on an interactive schematic of a cell. All data are freely available for download to facilitate large-scale analyses.

## Results

### The COMPARTMENTS web resource

**Figure 1.**
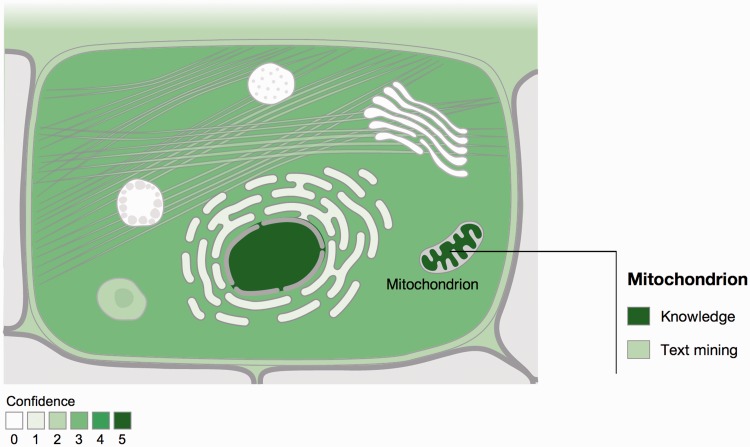
Visualization of localization evidence. When querying the database for a protein, its localization is visualized on a schematic of a cell. When the user hovers the cursor over a compartment, we also graphically summarize the types of evidence supporting this localization. The confidence of the evidence is color coded, ranging from light green for low confidence to dark green for high confidence. White indicates an absence of localization evidence.

COMPARTMENTS holds subcellular localization information for 22 705 human and 6696 yeast proteins, and covers also other eukaryotes such as fruit fly, mouse and *Caenorhabditis elegans*. When querying the database for a protein of interest, the user is presented with an interactive schematic of a cell. These figures are color coded according to the confidence of the evidence supporting each of the 11 (12 in case of plants) labeled compartments (Figure 1). Interactive tables provide the user with more fine-grained localization information and the source of the underlying evidence.

To provide a unified overview as described above, we map protein identifiers from the source databases to their corresponding identifiers in the STRING (*Search Tool for the Retrieval of Interacting Genes*) database (30), which for organisms in question come from Ensembl (31). We similarly map all cellular compartments to their respective GO cellular component terms (32). The labeled compartments are a subset of broad GO terms, much like GO Slims (33).

We further assign a confidence score to each piece of evidence to reflect that not all types and sources of localization information are equally reliable. To clearly signify that these should not be over-interpreted as probabilities, we use a scoring scheme that ranges from one star (lowest confidence) to five stars (highest confidence). The way that confidence scores are assigned varies between evidence channels as explained in the next section. The confidence scores are also the basis for the color coding of the figures (Figure 1): the higher the confidence, the darker the shading of the compartment.

### Evidence channels and sources

The evidence contained in COMPARTMENTS is logically partitioned into four channels of evidence. The first channel, called *knowledge*, is based on annotations from UniProtKB (1), MGI (2), SGD (3), FlyBase (4) and WormBase (5). We assign confidence scores to these annotations based on the associated GO evidence codes (34, 35), which encode whether the annotation is based on a peer-reviewed publication, an experimental data set or sequence similarity (see Methods section). The knowledge channel provides localization information on 16 864 human and 5909 yeast proteins.

HPA (36) is an ongoing effort to experimentally validate the tissue expression and subcellular localization for the entire set of human proteins. The latter data are captured by the *experiments* channel and currently contain information on 9306 human proteins. The confidence scores of this channel are based on the antibody validation scores provided by HPA (15) (see Methods section).

The third channel provides associations between proteins and subcellular localizations derived from automatic text mining of the abstracts in Medline. We used the dictionary of protein names from STRING (30) and created a dictionary of subcellular compartments from GO (see Methods section). We use a confidence scoring scheme, which is based on the fact that the more a protein and a cellular compartment are co-mentioned, the more likely the protein is to be localized to the compartment (see Methods section). The *text-mining* channel currently contains putative localizations for 15 304 human and 4144 yeast proteins.

Finally, the *predictions* channel contains precomputed results from two sequence-based prediction methods, namely the well-known WoLF PSORT (37) and the high-resolution version of YLoc (23, 24). Published benchmarks (21, 23) suggest that these methods are two of the best that cover many compartments, in particular for human proteins. Moreover, these and the other methods mentioned earlier were developed on overlapping training sets, and thus cannot be considered independent evidence. The primary reason for including only two methods is thus to not present the user with a large number of redundant predictions. We applied both methods to 22 523 human, 23 443 mouse, 22 938 rat, 14 076 *Drosophila melanogaster*, 20 158 *C. elegans*, 6697 *Saccharomyces cerevisiae* and 31 280 *A. thaliana* protein sequences from STRING 9.1 (30). The output scores from each tool were transformed to make them comparable with other evidence in the database (see Methods section).

**Figure 2.**
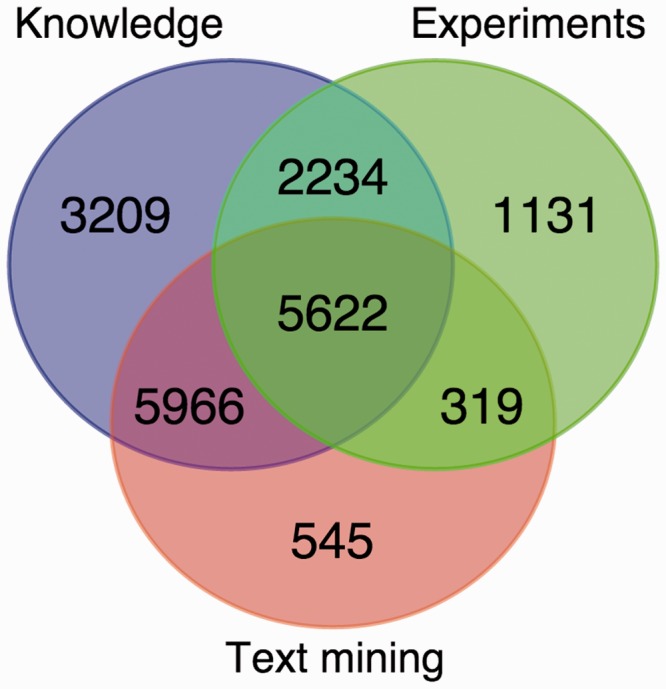
Overlap between the knowledge, experimental and text-mining evidence for human proteins. The Venn diagram shows the number of proteins with localization evidence from one or more of the three types of evidence. The two sequence-based prediction methods are not included as they are able to provide a prediction for any protein sequence.

The number of human and yeast proteins assigned to each of the 11 labeled compartments based on each of these evidence channels are summarized in Tables 1 and 2, respectively. The two sequence-based prediction tools both provide full coverage of the proteome and are therefore shown separately in the tables. For this reason, we also leave out the prediction tools in Figure 2, which shows the overlap in terms of human proteins assigned to at least one compartment by knowledge, experiments and text mining. This shows that integrating experimental and text-mining evidence increases the coverage by 11% additional human proteins. Even when more than one channel covers the same protein, this is not necessarily redundant information. Firstly, the same protein can localize to multiple compartments, and the two evidence channels may not provide support for the same localization. Secondly, when two channels support the same localization of a protein, they typically provide complementary evidence of interest to the user. This is also why full coverage of the sequence-based prediction tools does not make the other evidence channels redundant; if a protein is predicted to have a certain localization, it is still of interest to the user if this also is supported by experiments or literature.

**Table 1.** Overview of the localization evidence for human proteins

Compartment	Knowledge	Experi ments	Text mining	PSORT	YLoc
Nucleus	6082	5848	2288	9600	5335
Cytosol	2538	4872	577	9128	4630
Cytoskeleton	1843	1215	1257	134	–
Peroxisome	124	–	240	315	262
Lysosome	386	–	262	5	120
Endoplasmic reticulum	1382	151	656	281	178
Golgi apparatus	1250	814	348	64	313
Plasma membrane	4440	1271	1515	3681	3815
Endosome	170	–	88	–	–
Extracellular space	2267	–	1528	4331	1625
Mitochondrion	1156	924	793	2008	871

We counted protein–compartment associations separately for each of the 11 labeled compartments and for each evidence channel. The only exception is the predictions channel, for which we show the results from the two sequence-based methods (PSORT and YLoc) separately. Dashes denote compartments for which a channel or prediction method cannot provide evidence.

**Table 2.** Overview of the localization evidence for yeast proteins

Compartment	Knowledge	Text mining	PSORT	YLoc
Nucleus	2194	211	3870	1476
Cytosol	422	42	3242	1533
Cytoskeleton	231	108	44	–
Peroxisome	69	65	20	127
Vacuole	268	88	0	23
Endoplasmic reticulum	486	129	42	38
Golgi apparatus	236	75	12	57
Plasma membrane	457	135	775	350
Endosome	16	18	–	–
Extracellular space	94	69	302	624
Mitochondrion	1118	162	1486	422

For details refer to the footnote of Table 1.

### Benchmark of the text-mining pipeline

**Figure 3.**
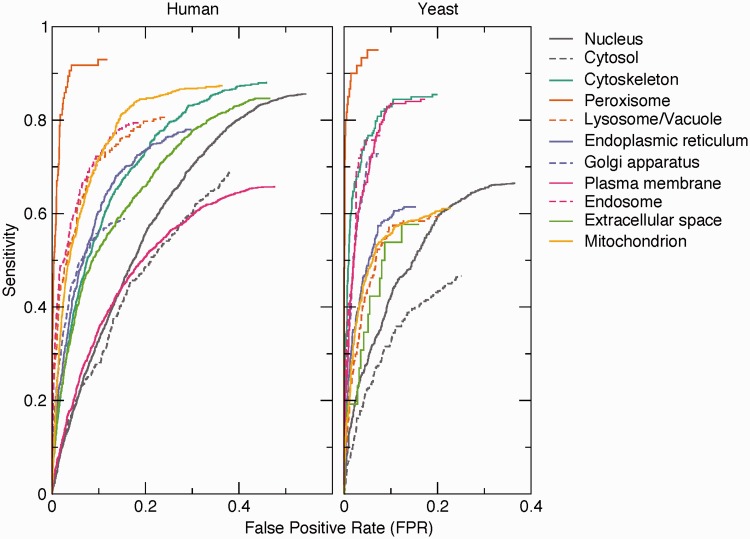
Benchmark of text-mining results. The performance of the text-mining pipeline on human and yeast proteins is shown as receiver operating characteristics (ROC) curves for each of 11 compartments. The curves do not intercept sensitivity = 1.0 and FPR = 1.0 because many of the protein–compartment pairs in the benchmark set are never found mentioned together in Medline, for which reason they have no text-mining score.

To assess the quality of the pairs extracted by text mining, we compared them against a benchmark set of 9764 human and 3834 yeast proteins having 12 232 and 4530 high-confidence localization annotations, respectively. The benchmark set is derived from the evidence in the knowledge channel (see Methods section). This shows that the method works well on the majority of the compartments (Figure 3). The exceptions include the nucleus and—in case of human—the plasma membrane. The false positives for these compartments are predominantly because of functional associations captured by co-mentioning. For example, a protein involved in signal transduction can easily be functionally associated with both the plasma membrane and the nucleus without being localized to either. The method also shows poor performance for the cytosol because of the experimental difficulty to distinguish proteins in the cytosol from those in, for example, vesicles. Consequently, many cytosolic proteins are conservatively annotated to the cytoplasm instead of the cytosol.

### Linking compartments by overrepresentation of shared proteins

**Figure 4.**
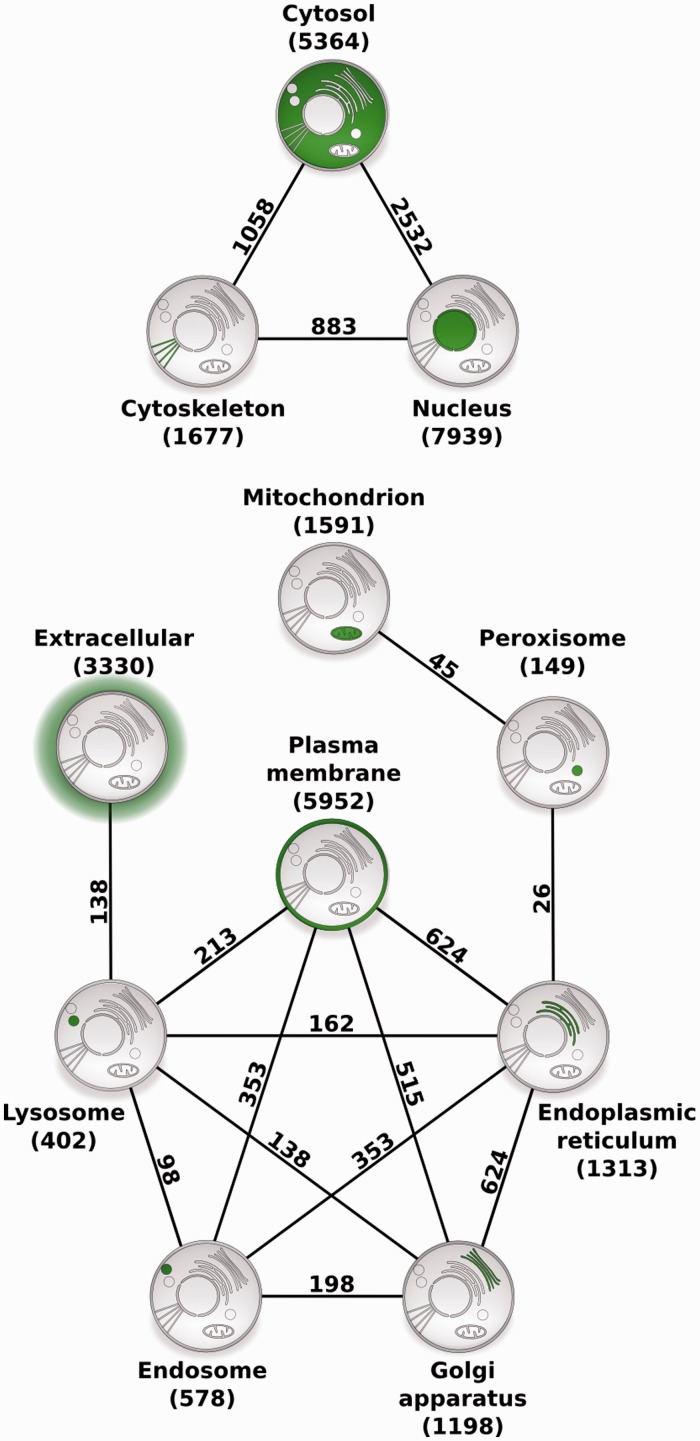
Compartment relationships derived from shared proteins. Illustrating the usefulness of COMPARTMENTS for global analysis of protein localization, we studied relationships between compartments. Each node represents a single compartment, which is highlighted in green. The number of proteins in the compartment is shown in parenthesis. We show an edge between two compartments whenever they share more proteins than expected at random (false discovery rate <0.1%). The number of proteins co-localized to the two compartments is shown next to the edge.

To illustrate the usefulness of COMPARTMENTS for large-scale studies, we identified pairs of compartments that share a statistically significant number of human proteins (Figure 4 and see Methods section). Notably, there were no borderline cases–all pairs of compartments were either highly significant after controlling for multiple testing or they were not even significant before correction. The two compartments that share the most proteins are the cytosol and the nucleus, both of which also share many proteins with the cytoskeleton. Most of the remaining intracellular compartments form a highly connected network, except the extracellular space, the mitochondria and the peroxisomes.

## Discussion

The COMPARTMENTS resource unifies complementary evidence on protein localization from curated knowledge, high-throughput experiments, text mining and sequence-based prediction methods. We go beyond merely integrating many sources of evidence into a single database by mapping all pieces of evidence onto the same set of identifiers and carefully assigning them comparable confidence scores. We derived these through a combination of manual inspection of each evidence source, a previous study of the reliabilities of GO evidence codes (38), the benchmark results for the text-mining pipeline and score distributions for the sequence-based prediction methods.

The primary aim of COMPARTMENTS web interface is to provide the user with a simple overview of the localization of a protein of interest without losing the connection to the underlying evidence. The overview is provided through a schematic of a cell, which is color coded based on the strongest evidence supporting each compartment. This visualization is interactive and allows the user to see which evidence channels support a particular compartment and how strongly it supports. This directly informs the user about which of the tables below contain further details about the origin of the evidence. For the knowledge and experiments channels, the tables link out to the external databases from which the evidence was obtained. For text mining, the table gives access to an abstract viewer that shows the abstracts in which the protein and localization are co-mentioned, highlighting the terms that were recognized.

Demonstrating the usefulness of COMPARTMENTS for large-scale analyses, we derived a network of compartments, which is highly consistent with established knowledge on protein trafficking. The strong association between the cytosol and the nucleus is unsurprising, as nucleocytosolic protein transport is a well-established regulatory mechanism (39). Both compartments also share many proteins with the cytoskeleton, most of which are involved in processes such as centrosome organization, chromosome segregation and nuclear division, which is consistent with the highly dynamic interplay between these compartments during mitosis (40). We further found that peroxisomes are related to the endoplasmic reticulum and to the mitochondria by proteins mainly involved in fatty acid metabolic and lipid biosynthetic processes. In contrast to the well-studied metabolic cooperation between the peroxisomes and the endoplasmic reticulum (41, 42), the connection to mitochondria was only recently discovered (43, 44), and the underlying mechanistic link is not yet fully understood (45). Proteins shared between the plasma membrane, endosomes and lysosomes, and those shared between lysosomes and the extracellular matrix are mainly involved in immune response and phagocytosis, which are related to the endocytic trafficking pathway (46, 47). The links between the endoplasmic reticulum, Golgi apparatus and plasma membrane reflect the exocytotic pathway (48). Lastly, cross talk between these major trafficking pathways between intracellular organelles (49) is captured by the connections between the Golgi apparatus, endosomes and lysosomes. Because COMPARTMENTS uses the same protein identifiers as the STRING database (30), it also facilitates large-scale analysis of protein localization in the context of interaction networks.

COMPARTMENTS is the first resource to integrate subcellular localization evidence from manually curated annotations, high-throughput screens and sequence-based predictions with automatic text mining for all major model organisms. To avoid the common problem of bioinformatics databases not being maintained, we have from the beginning designed the resource to be automatically kept up-to-date with the constant changes in source databases and literature. We address the challenge of making it easy for users to comprehend the heterogeneous evidence by projecting it onto a common reference both in terms of protein and compartment identifiers and in terms of reliability scores. This is complemented by the web interface, which provides an intuitive, interactive graphical overview of the unified evidence and simple tables with more detailed information, including links to the original sources. We also make the unified evidence available as bulk download files to facilitate large-scale computational studies of protein localization and integration with omics data sets.

## Materials and Methods

### Visualization of protein subcellular localization

For visualization purposes, we selected a set of commonly used localizations, including the cytosol and all major organelles. Each of these represents a GO term, and all evidence for more fine-grained localizations is projected onto these through is_a and part_of relationships. In case of multiple lines of evidence for the same localization, we always select the strongest. We subsequently present the evidence by color coding a schematic of a cell. We have developed separate figures for animal, fungal and plant cells to account for differences in their cell structure; for example, animal cells have no cell wall, and only plants have chloroplasts.

### Assembly of the knowledge and experiments channels

We imported subcellular localization annotations from comments and database cross-reference fields of UniProtKB. We map these to the corresponding Ensembl identifiers using the STRING alias file (30) and GO terms using the UniProtKB controlled vocabulary of subcellular localizations. For *S. cerevisiae*, *C. elegans*, *D. melanogaster* and *Mus musculus*, we imported cellular component GO annotations from their respective model organism database (2–5).

For the knowledge channel, we assigned the highest score of four stars for annotations with the following evidence codes: CURATED, IDA, TAS and NAS. We assigned three stars to the evidence codes PROBABLE, EXP, IPI, IMP, IGI, IEP, ISS, ISO, ISA, ISM, IBA, IBD, IKR, IMR, IRD and IC. We assigned two stars to the less reliable evidence codes POTENTIAL, IGC and IEA, while BY SIMILARITY, RCA and NR are assigned only one star. Because we consider some sources to be more reliable than others, we upgraded annotations from UniProtKB and the model organism databases by one star, resulting in a maximum score of five stars for the knowledge channel.

We also imported subcellular localization data from the Human Protein Atlas (HPA) (15, 50), which uses Ensembl identifiers, and manually mapped their locations to the corresponding GO terms. HPA uses two scoring schemes to classify the quality of its data. When a protein has been stained using two or more antibodies, HPA provides a *reliability score* based on the similarity of the staining patterns obtained with the different antibodies and the agreement with published literature. This scale has four levels of reliability: high (four stars), medium (three stars), low (two stars) and very low (one star). When only a single antibody has been used for staining, we instead make use of the *validation score* provided by HPA. This scale has three levels: supportive (three stars), uncertain (one star) and non-supportive (not imported).

### Text mining of Medline abstracts

We used the protein dictionary from STRING 9.1 (30) and created a dictionary of names of subcellular localizations from the cellular component terms of the GO (32). To improve the protein dictionary, we discarded protein names that conflict with names of GO terms. Furthermore, we blocked frequently occurring ambiguous names, such as acronyms, thereby greatly improving the precision. This was done through manual inspection of all protein and localization names giving rise to >2000 matches in Medline.

We matched these dictionaries against all Medline abstracts using an efficient named entity recognition engine described elsewhere (51). To score the co-occurring proteins and localizations, we used the text-mining scoring scheme of STRING 9.1 (30), which is a weighted count [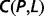] for each pair of protein 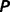 and for localization 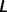:

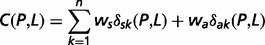

where *n* is the number of abstracts, 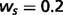 and 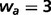 are the weights for co-occurrence within the same sentence and within the same abstract, respectively. If 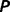 and 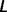 are mentioned together in a sentence or in abstract 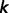, the delta functions 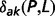 and 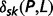 are 1, and 0 otherwise. Thus, an abstract that mentions *P* and *L* in the same sentence will give a score contribution of 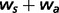, whereas an abstract that mentions them in different sentences will give a score contribution of 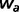 only. The co-occurrence score [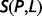] is defined as follows:

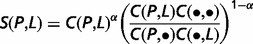

where 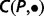, 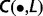 and 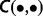 are the sums over localizations paired with protein 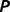, over all proteins from the same organism paired with localization 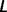 and over all pairs of proteins from the same organism and localizations, respectively. The weighting factor 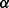 is 0.6. All parameters in the scoring scheme (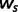, 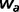 and 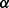) were optimized to maximize the agreement between protein–protein co-occurrence scores and KEGG pathways (30).

The text-mining score depends on number of pairs identified in Medline abstracts, which changes as Medline grows. We, therefore, convert the scores into z-scores [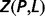] to get a more robust measure. The observed distribution is a mixture of two, one from low-scoring random pairs and second from high-scoring biologically meaningful pairs. The former is modeled as a Gaussian where the mean is equal to the mode of the observed distribution, which empirically coincides with the 40th percentile. The variance of the background is estimated from the difference between the 20th and the 40th percentiles. The final confidence score, stars, is the z-score/2, limited to a maximum of four.

### Construction of text-mining benchmark set

We constructed a high-quality benchmark set based on the knowledge channel. The positive examples are pairs of proteins and compartments supported by five-star evidence. The negative examples are pairs of proteins and compartments for which there is no evidence suggesting that the protein is in the compartment and five-star evidence for the protein being in a different compartment. The compartments considered for the benchmark set are the 11 subcellular localizations used in the overview figure, and all evidence for more specific localizations have been backtracked to this level. The benchmark set is available for download from the COMPARTMENTS web resource.

### Scoring of sequence-based predictions

The WoLF PSORT and YLoc-HighRes methods were selected for prediction of subcellular localization. We precomputed predictions for the entire set of protein sequences for human, mouse, rat, *D. melanogaster*, *C. elegans*, *S. cerevisiae* and *A. thaliana* in STRING 9.1. We converted all scores to stars to make them comparable with other evidence types; the maximum number of stars that can be assigned to a sequence-based prediction is three. This ensures that prediction scores cannot exceed the scores of reliable manual annotations, experiments or text mining.

PSORT (37) predicts localization based on various sequence-derived features such as sorting signals, binding domains and amino acid composition. These are used by a weighted 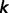-nearest neighbor classifier. The output scores 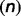 roughly correspond to the number of the 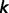 nearest neighbors from the training set that are annotated with each localization. We convert these scores to stars 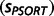 using the following formula:

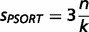



YLoc (23) is a naïve Bayes classifier that uses features similar to those of PSORT combined with GO annotations of close homologs. We found that most of the posterior probabilities from YLoc are close to either 0 or 1. To differentiate between the probabilities close to 1 when converting them to stars, we transform them using the following heuristic function:

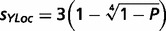

where *s_YLoc_* is the stars derived from a YLoc prediction, *P* is the prediction probability that the protein is localized in the given compartment. (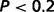 is ignored). This formula ensures that probabilities close to 1 become distinguishable when converted to stars: 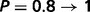 star, 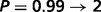 stars, 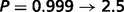 stars and 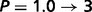 stars.

### Statistical analysis of compartments sharing proteins

From the unified data set, we extracted localization information on human proteins with more than two stars to disregard weak text-mining and prediction evidence. The retrieved data set comprised 18 692 unique human proteins with 29 493 links to compartments: 20 021 were supported by curated knowledge, 4841 by high-throughput experimental evidence, 1468 by text mining and 15 788 by sequence-based predictions. We counted the number of proteins shared between any two compartments. To assess if this is higher than expected, we compared the counts to a null model that assumed no correlation between any compartments. To this end, we generated 1 000 000 random data sets in which links between proteins and compartments were permuted, thereby preserving the number of links per protein and per compartment. We computed a *P*-value for each pair of compartments as the fraction of random data sets resulting in a count greater than or equal to the observed count. Finally, we defined the statistically significant compartment pairs by imposing a false discovery rate of 0.1% using the Benjamini–Hochberg method (52).
